# A question of data quality—Testing pollination syndromes in Balsaminaceae

**DOI:** 10.1371/journal.pone.0186125

**Published:** 2017-10-16

**Authors:** Stefan Abrahamczyk, Sissi Lozada-Gobilard, Markus Ackermann, Eberhard Fischer, Vera Krieger, Almut Redling, Maximilian Weigend

**Affiliations:** 1 Nees-Institute for Biodiversity of Plants, University of Bonn, Meckenheimer Allee 170, Bonn, Germany; 2 Institute of Biochemistry and Biology, University of Potsdam, Maulbeerallee 1, Potsdam, Germany; 3 Institute for Integrated Natural Sciences–Biology, University of Koblenz-Landau, Universitätsstraße 1, Koblenz, Germany; Institute of Botany Chinese Academy of Sciences, CHINA

## Abstract

Pollination syndromes and their predictive power regarding actual plant-animal interactions have been controversially discussed in the past. We investigate pollination syndromes in Balsaminaceae, utilizing quantitative respectively categorical data sets of flower morphometry, signal and reward traits for 86 species to test for the effect of different types of data on the test patterns retrieved. Cluster Analyses of the floral traits are used in combination with independent pollinator observations. Based on quantitative data we retrieve seven clusters, six of them corresponding to plausible pollination syndromes and one additional, well-supported cluster comprising highly divergent floral architectures. This latter cluster represents a non-syndrome of flowers not segregated by the specific data set here used. Conversely, using categorical data we obtained only a rudimentary resolution of pollination syndromes, in line with several earlier studies. The results underscore that the use of functional, exactly quanitified trait data has the power to retrieve pollination syndromes circumscribed by the specific data used. Data quality can, however, not be replaced by sheer data volume. With this caveat, it is possible to identify pollination syndromes from large datasets and to reliably extrapolate them for taxa for which direct observations are unavailable.

## Introduction

For more than 250 years scientists have been aware that flowers are morphologically adapted to their pollinators and vice versa [1,2]. Darwin [3] was the first to place this interaction into an evolutionary context and inspired others to study pollination ecology [4–9]. Based on these studies, Vogel [10] summarized and Fægri and van der Pijl [11,12] further developed the classical, category-based concept of pollination syndromes: a combination of flower traits such as flower architecture, coloration, scent and reward that evolved by interactions with a specific group of animal pollinators. Since then, a large number of studies have applied this concept to provide a mechanistic explanation for floral diversity, and to infer pollinator groups for plant species for which observational data were missing [13–16]. Several studies found support for the concept of pollination syndromes [17–22]. However, the general applicability of the concept has also been questioned several times, mainly because many plant species are known to be pollinated by a wide range of animals from different pollinator groups [23–28]. Also, some plant species can not clearly be assigned to any individual syndrome because they show atypical characters, or a combination of characters typical for more than one syndrome [29]. Thus, individual floral traits may be entirely under the selection pressure of a secondary pollinator since the primary pollinator is able to effectively deal with a wide trait range [30]. It has therefore been argued that applying the classical, category-based syndrome concept does not reliably circumscribe the diversity of floral phenotypes and is unreliable in predicting major pollinator groups [24,29,31–32]. Additionally, its predictive power might differs across plant families [28,29]. Nevertheless, Fenster et al. [13] partly resolved this debate arguing that plant species pollinated by animals from two different pollinator groups may well have a specialized pollination syndrome if the pollinators are functionally homogeneous, forming a pollinator guild, for example plant species pollinated by butterflies and long-proboscid flies or species pollinated by syrphid flies and short-tongued bees [13]. Additionally, several plant species are known to have a primary and a secondary pollinator guild, which may diverge in abundance and/or efficiency [27,33,34]. Stebbins [35] proposed that convergent floral features are adapted to those pollinators visiting flowers most frequently and effectively, which implies that some species may well be visited and sometimes pollinated by animals that do not fit the syndrome [33] without necessarily contradicting the concept of pollination syndromes. This latter phenomenon is apparently more common in temperate regions, and secondary pollinator guilds may often represent the ancestral guilds [33].

**Fig 1:**
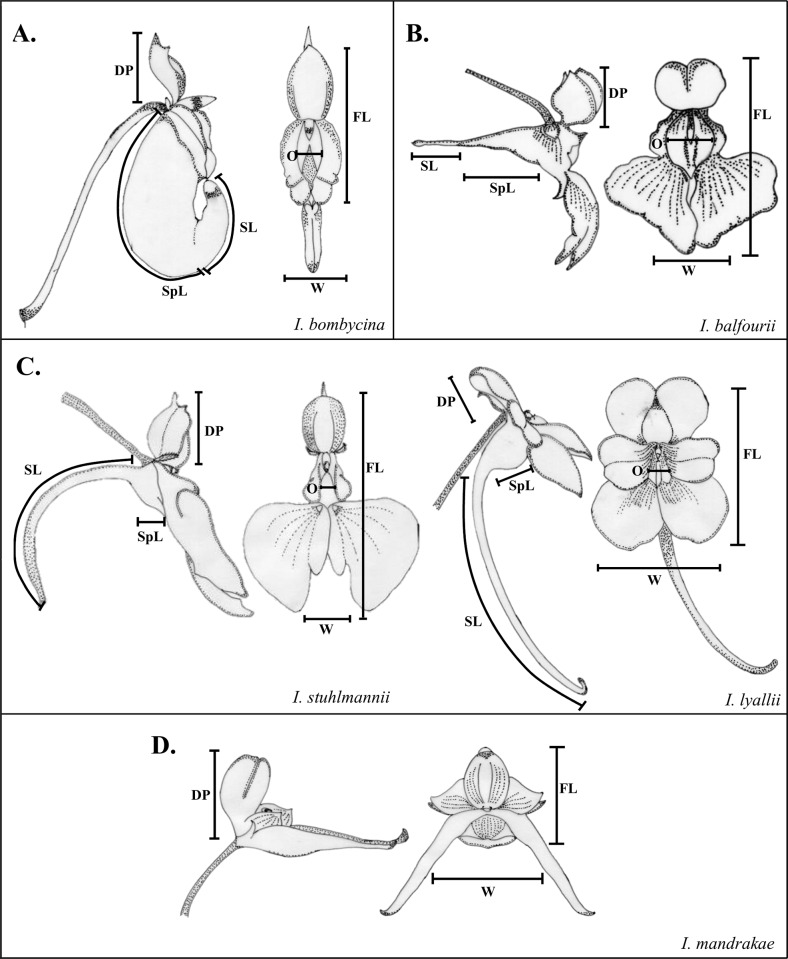
Morphometrical measurements conducted in the different flower types in Balsaminaceae: Lateral view: DP: dorsal petal, SL: Spur length, SpL: Spur carrying sepal length. Frontal view: O: Width of flower opening, W: Flower width, FL: Flower length. Drawings: S. Lozada-Gobilard.

The Balsaminaceae (Ericales), with the two genera, *Hydrocera* (1 sp.) and *Impatiens* (>1000 spp.), are a singularly suitable group for studying pollination syndromes. They are most diverse in montane forests of the Old World tropics and subtropics [36]. The genus *Impatiens* is characterized by an outstanding diversity in flower shape, size and coloration [37] and has thus been characterized as the "dicots counterpart of orchids" [38]. The flowers are zygomorphic and pentamerous. The five sepals are morphologically disparate: one pair of them usually strongly reduced, one pair regularly developed and one petaloid lower sepal, modified into a nectar-containing spur. The corolla is also strongly modified. The adaxial petal is usually hood-like petal and the other four petals are united into two lateral pairs (compare Fig 1). Grey-Wilson [37] and Akiyama et al. [39] classified different architectural flower types. However, this typological classification is problematical due to a wide range of intermediate flower architectures. The tremendous diversity in flower architecture within *Impatiens* has been associated with the presence of a wide range of different pollinator groups [37]. Field studies identified five main pollinator groups: birds, moths, bees, butterflies and flies [40–43]. However, a connection between flower architecture, signal and reward versus pollination syndromes has not so far been demonstrated conclusively.

Categorical data have mostly been used for testing for pollination syndromes in several studies, which generally found at best weak support for the concept [e.g. 16,25,29]. The power of such analyses is limited, and studies rigorously testing the existence of pollination syndromes, applying quantitative morphometry, signal and reward data would be a way forward in evaluating the syndrome concept. The aim of the present study is to test for convergent adaptations to groups of pollinators within the Balsaminaceae considering the three central dimensions of the plant-pollinator interaction (flower architecture, signal and reward) and to compare the results of two sets of analyses, applying quantitative respectively categorical data. Both data sets will be explored by cluster analyses and the results will be evaluated and interpreted in combination with observational pollinator data to intersubjectively group species into pollination syndromes. Based on our results we aim at addressing the following two questions:

1. Do quantitative or categorical flower morphometry, signal and reward data define well-supported clusters in Balsaminaceae?
2. Do the clusters retrieved reflect individual pollination syndromes distinguished by characteristic flower traits?

## Materials and methods

### Sampling

Flower morphometry, signal and reward data from 86 species (85 species of *Impatiens* and *Hydrocera triflora*) from the Balsaminaceae collection at the Bonn University Botanical Gardens were compiled 2012 to 2015 (S1 Table). Usually, a single accession (= one genetic individual) was investigated for each taxon. Three different accessions of *Impatiens* aff. *niamniamensis* were included into our sampling, since these accessions appear to represent three different (currently unresolved) taxa (E. Fischer, pers. obs.). A standardized methodology was implemented to take all measurements under controlled conditions in the absence of flower visitors. We measured six different functional, morphometrical traits, quantifying ten flowers per trait and species: spur length, spur-carrying sepal length, total flower length, width of flower opening, flower width and dorsal petal length (Fig 1). Flower signal, i.e. frontal and lateral display size, total display size as well as the ratio between frontal and lateral display were obtained by taking flower photos frontally and laterally in a standardized way and analysing the pixel number of the flowers using Adobe Photoshop CS6 (Adobe Systems Software Ireland Ltd).

Nectar of 25 flowers per species was sampled between 9 a.m. and 1 p.m. to determine the nectar volume per flower. The only species likely pollinated by night-active insects, (*Impatiens sodenii*, probably pollinated by hawk moths) produces relatively large amounts of nectar during the day, so sampling was uniformly conducted in the same time period for all taxa. All flowers sampled were mid-anthetic (late staminate or early pistillate phase) to cater for possible differences in nectar production during anthesis. Nectar was extracted from the spur with microcapillaries (Hirschmann Laborgeräte, 0.5, 1, 10 μl). In flowers without spur, nectar is secreted on the lower sepal and was sampled from there. In some species we were unable to take up nectar with the capillaries. In these species the absence of nectar was verified by testing for the presence of glucose with glucose-testers (Combur Test HC). Total sugar concentration was measured with a handheld refractometer (neoLab-Handrefraktometer Universal; 0–80% Brix). The amount of sugar per flower was calculated following Dafni et al. [44].

Known pollinators from the natural range of the species studied were compiled from the literature (Scholar Google and ISI Web of Science, search tags “*Impatiens*”, “Balsaminaceae”, and “*Hydrocera*” in combination with “pollinator” or “pollination”). A list of pollinator references is provided in S2 Table. Following Rosas-Guerrero et al. [33] we only considered surveys conducted under natural conditions recording all observed pollinators. Only animals contacting the reproductive organs and visiting flowers also in the carpellate phase were considered as pollinators. For categorizing the individual functional pollinator guilds, we followed Fægri and van der Pijl [12] and Fenster et al. [13].

### Statistical analyses

Correlations between flower morphometry, signal and reward traits were investigated by Spearman correlation tests to understand how the different flower traits are related to each other. In order to evaluate the existence of pollination syndromes within Balsaminaceae we generated one Euclidean distance matrix for all twelve untransformed flower morphometry, signal and reward traits and used it to conduct a Cluster analysis applying the UPGMA algorithm (Unweighted Pair Group Method with Arithmetic Means). In a first step, we included only those 21 Balsaminaceae species into the cluster analysis for which we had independent pollinator observations (S2 Table). In a second step, we repeated this analysis for the complete dataset (86 species) and added independent pollinator information as well as the syndrome categorization of Grey-Wilson [37] and Erpenbach [45] to the remaining species. Then, we compared the cluster pattern obtained from the small versus the large data set. To additionally verify our results with a different method we then performed multiscale bootstrap resampling to cluster the species, applying an Euclidean distance matrix, the “complete” clustering method and 1000 bootstrap runs in a third step.

Finally, we tested for homogeneity of variance in the morphometry, signal and reward trait data. Since we neither received homogeneity of variance with untransformed nor with transformed data we conducted Kruskal-Wallis tests for multiple comparisons with untransformed data to analyse which morphometry, signal and reward traits differ between individual clusters of the cluster analysis containing 86 species.

To compare the impact of using quantitative versus categorical data on the results of the cluster analysis we categorized our quantitative data. Trait values smaller than the median were categorized as “low” and the values higher than the median as “high”. Only for traits absent in one or more species we added a third category “not existing” (S3 Table). The categorized data were used to generate a Bray-Curtis distance matrix. Based on this distance matrix we then conducted a second Cluster Analysis applying the UPGMA algorithm. The congruence between the clusters obtained from quantitative respectively categorical data was investigated by conducting a Mantel test.

All analyses were performed in R i386 (version 3.0.2 [46]). Multivariate analyses were conducted using the vegan package [47] and multiscale bootstrap resampling was performed with the pvclust package [48].

## Results

**Fig 2:**
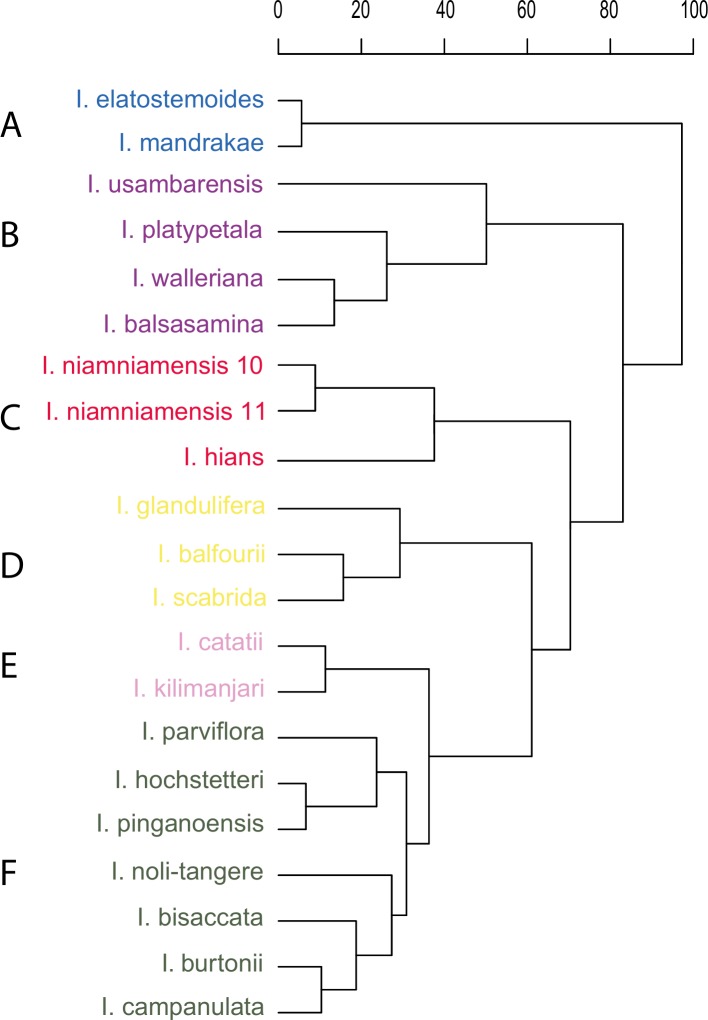
Cluster analyses generated from a PCA based on quantitative flower morphometry, signal and nectar traits for those 21 Balsaminaceae species, for which independent pollinator observations exist (S2 Table): **A:** generalist fly; **B:** butterfly; **C:** bird 1; **D:** large bee; **E:** bird 2; **F:** non-syndrome (containing several syndromes).

**Fig 3:**
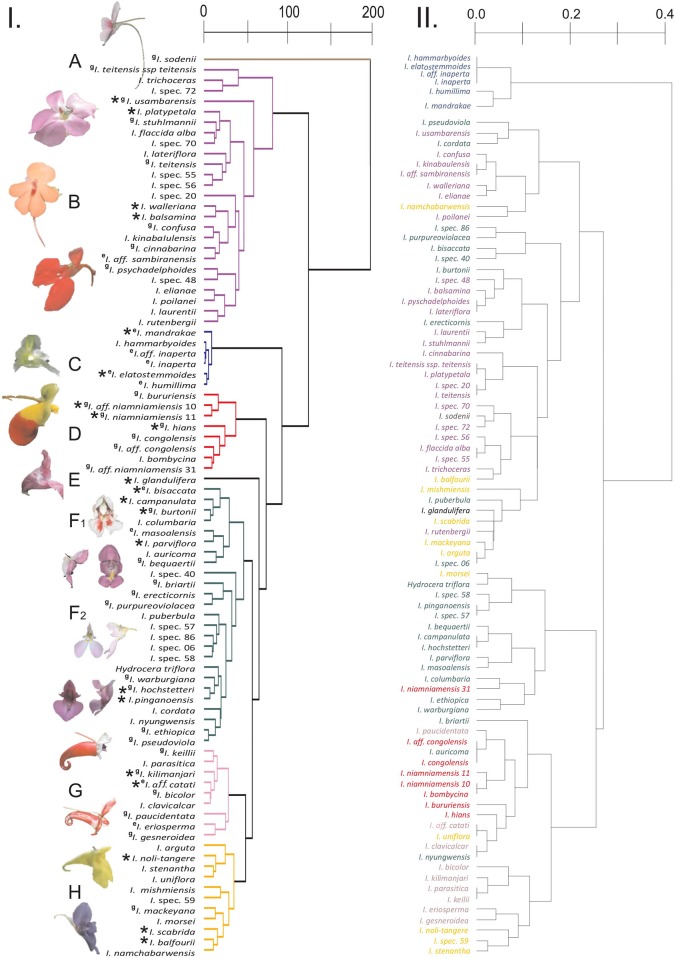
Cluster analyses generated from distance matrices (I.) based on quantitative and (II.) based on categorical flower morphometry, signal and nectar traits. Pollination syndromes are assigned to the clusters of I. based on syndrome assignments by Grey-Wilson [37] (^g^) and Erpenbach [45] (^e^) as well as pollinator observations for individual species (*) from the literature: **A:** hawkmoth; **B:** butterfly; **C:** generalist fly; **D:** bird 1; **E:** large bee (Apidae); **F1** and **F2:** non-syndrome (containing several syndromes); **G:** bird 2; **H:** large bee (Apidae; tribes Anthophorini, Apini, Bombini). Coloration of the species in II. is based on the coloration of the clusters in I. In contrast to Grey-Wilson [37] we assigned the hawkmoth syndrome to *Impatiens sodenii*, based on the 12.8 cm long spur and the largest frontal display size of all analysed species. In contrast to Erpenbach [45] we assigned the generalist fly syndrome to *I*. *inaperta* and *I*. aff. *inaperta* since we analysed only open, non-cleistogamous flowers.

Even though we used widely different functional traits, many of them are highly correlated (S4 Table). The Cluster Analyses including 21 respectively 86 species retrieved very similar clustering patterns (Figs 2 & 3I) retrieving six clusters and in case of the large data set two individual Balsaminaceae species outside the clusters (A: *Impatiens sodenii* and E: *I*. *glandulifera*). Five of the clusters retrieved can be clearly assigned to pollination syndromes based on published pollinator observations and syndrome assignments. These syndromes are: butterfly, generalist fly (Diptera, morphologically not highly adapted to feed on nectar), bird 1, bird 2, and large bee (Apidae; tribes Anthophorini, Apini, Bombini). Cluster F includes species pollinated by small to medium-sized insects, including small bees, syrphid flies, long-proboscid flies, butterflies and can thus not clearly be assigned to a single functional pollinator guild. We therefore call this cluster the non-syndrome cluster. However, within this cluster the pollinator guilds of the individual plant species form two largely ecologically homogeneous sub-clusters (Fig 3I): one containing mainly species pollinated by small bees and syrphid flies (sub-cluster F1) and a second containing mainly species pollinated by butterflies and long-proboscid flies (sub-cluster F2).

The clustering result applying multiscale bootstrap resampling looked similar to the result of the traditional cluster analysis. All syndrome clusters received by the traditional analysis were again retrieved by multiscale bootstrap resampling. In addition, two large bee and butterfly clusters appeared each, *Impatiens glandulifera* and *I*. *balfourii* were part of the butterfly clusters and *I*. *puberula* was part of the bird 2 cluster. Further, the “bird 2” cluster was not retrieved as “monophyletic”. All clusters had bootstrap support of >80%, which supported the validity of the ecologically slightly more homogeneous clusters we obtained from our traditional cluster analysis. Therefore, we focus on the result of the traditional cluster analysis for the discussion and provide the results of the multiscale bootstrap resampling in S1 Fig.

**Fig 4:**
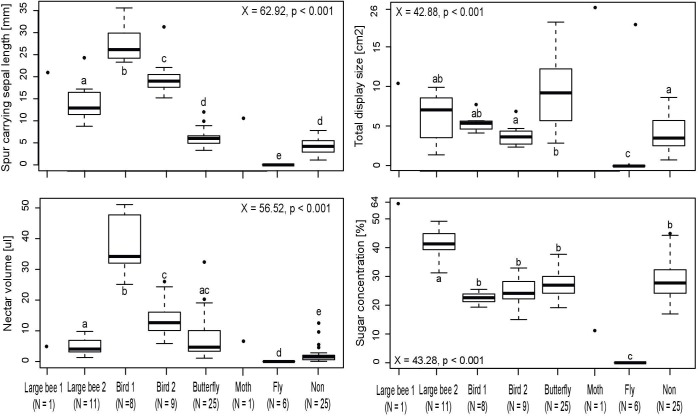
Kruskal-Wallis tests for multiple comparisons of **A:** spur carrying sepal length, **B:** total display size, **C:** nectar volume and **D:** sugar concentration between pollination syndromes. Note that Large bee 1 (= *Impatiens glandulifera*) and Moth (= *I*. *sodenii*) was not included into the analyses due to too small sampling sizes.

The Kruskal-Wallis tests for multiple comparisons showed that each cluster of the traditional cluster analysis can be distinguished by a characteristic set of flower morphometry, signal and/or reward traits (Fig 4, S2 Fig). The species of the bee 2 syndrome cluster have the widest flower openings and produce nectar with the highest sugar concentration. The species of the two bird syndrome clusters exhibit the largest spur-carrying sepal lengths. However, the two bird clusters differ in spur-carrying sepal length as well as in nectar volume and sugar amount. The species of the butterfly syndrome cluster are characterized by the longest spurs and the largest frontal display sizes in comparison to all other syndrome clusters except moth (*Impatiens sodenii*). The species of the non-syndrome cluster have the shortest spur-carrying sepals and the second lowest nectar and sugar production. Only the taxa retrieved in the fly syndrome cluster exhibit significantly lower values in these two traits. Finally, the species of the fly syndrome cluster have the smallest flowers and produce very little to no nectar at all. *Impatiens sodenii* (moth syndrome) has by far the longest spur, the largest frontal display size and the lowest nectar sugar concentration of all spurred species. *Impatiens glandulifera* (bee 1 syndrome) has the longest spur-carrying sepal of all insect-pollinated species and the highest nectar sugar concentration.

The cluster analysis based on categorical data shows some clustering of species, similar to the cluster analysis using quantitative data, e.g., some species pollinated by generalist flies or smaller birds. However, the clusters retrieved correspond poorly to clear pollination syndromes (Fig 3II). Individual species showing a clear bird, butterfly or large bee syndrome cluster with taxa displaying different other syndromes. The species of the “non-syndrome” are distributed all over the clusters, even within the bird syndrome. The Mantel test revealed a significant (p<0.001) correlation between the clusters received in the categorical versus quantitative analyses with a relatively low R-value (0.426).

## Discussion

Balsaminaceae are famous for their enormous diversity in flower architecture as well as an extraordinary floral complexity. Nevertheless, functional flower morphometry, signal and reward traits are highly correlated, indicating a concerted evolution of these traits. Both cluster analyses based on quantitative flower morphometry, signal and reward data, including 21 respectively 86 species, resulted in very similar clustering patterns of species indicating a convergent adaptation to groups of pollinators. In both cases six clusters (Fig 3I) and in case of the large data set additionally two individual species (*Impatiens sodenii* and *I*. *glandulifera*) are segregated. The clusters are characterized by unique flower traits (Fig 4, S2 Fig). Five of these clusters as well as both singlet species can be clearly linked to defined pollination syndromes based on independent pollinator observations and published syndrome assignments. These results clearly show that our study, using quantitative data on flower morphometry, signal and reward traits is able to confirm the existence of distinct, well defined pollination syndromes within Balsaminaceae.

Somewhat surprisingly, the large bee-pollinated *Impatiens glandulifera* [7] is retrieved separate from the large bee cluster, and bird-pollinated species are found in two distinct clusters. The raw data underscore that the long, tube-shaped spur-carrying sepal and the very high nectar sugar concentration distinguish *I*. *glandulifera* from all other large-bee-pollinated species. Additionally, pollen appears to be an important secondary reward in *I*. *glandulifera* (Lozada-Gobilard; pers. obs.), which makes it highly attractive to bumblebees in both its native range in the Himalaya and in Europe [49–51]. Thus, the divergent reward system is correctly reflected in the results of the cluster analysis. The two clusters with bird-pollinated species appear to represent different sub-syndromes of bird pollination. Cluster D (Fig 3) contains species with the longer spur-carrying sepals (Fig 1) producing more nectar and visited by large sunbirds [43], whereas the species in cluster G are smaller-flowered, have less nectar and are visited by smaller sunbirds and probably sunbird asities (Philepittidae [45,52]). Thus, by using quantitative traits we can not only test for pollination syndromes, but may also resolve previously non-recognized sub-syndromes characteristic of these clusters.

**Fig 5:**
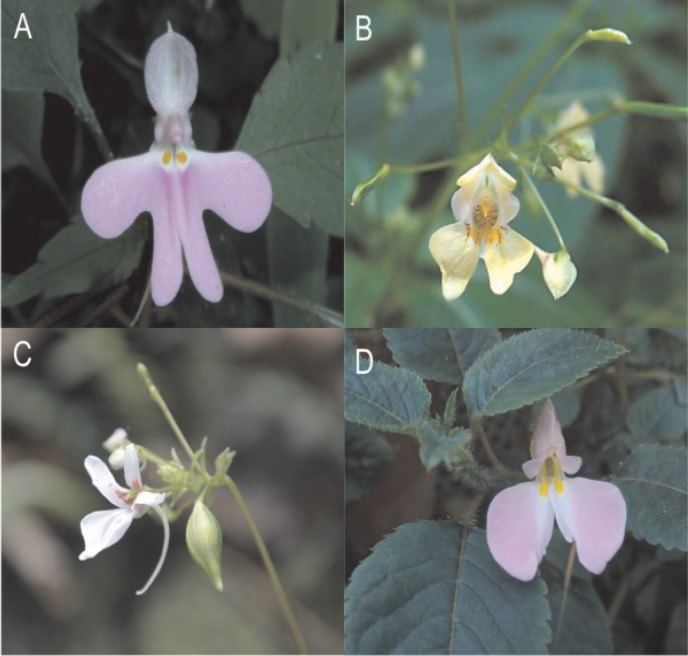
*Impatiens* species belonging to the non-syndrome cluster but having well defined, functionally homogeneous pollinator guilds; **A:** *I*. *hochstetteri*, **B:** *I*. *parviflora*, **C:** *I*. *pinganoensis*, **D:** *I*. *burtonii*; photos of *I*. *hochstetteri* by JMK, wikipedia.org, and *I*. *parviflora* by ArtMechanic wikipedia.org.

Cluster F (Fig 3), however, does not correspond to any recognizable pollination syndrome. It comprises insect-pollinated species, characterized by relatively small flowers, short spur-carrying sepals and a low nectar volume. Some species appear to belong to clearly circumscribed, classical pollination syndromes: butterflies and long-proboscid flies as pollinators of *Impatiens hochstetteri* [53] or short-tongued bees and syrphid flies as pollinators of *Impatiens burtonii* ([54] Fig 5) represent functionally homogeneous pollinator guilds. The cluster also contains *Impatiens campanulata*, the only generalist species pollinated by both bees and butterflies [41]. Functionally, the species of this cluster are far from representing a single pollination syndrome–it is a “non-syndrome“. Rather than being “pulled” together by sharing pollinator-specific traits, they are “pushed” together by not sharing the traits characterizing the syndromes retrieved in the other five clusters: The constituent taxa are not (sufficiently) differentiated from each other in the traits quantified in our particular dataset to be broken down into “real” syndromes, while being different enough from the taxa in the other clusters not to be pulled into any one of them. The taxa of the “non-syndrome” evidently correspond to several different pollination syndromes, differentiated in traits not here analysed.

The presence of the non-syndrome cluster thus shows the limitations of our data set. Its existence further underlines the complex nature of pollination syndromes, encompassing a large number of traits in morphometry, signal and reward [10], only a subset of which is reflected in our data set. Therefore, our results highlight the importance of using functional traits representing actual plant-pollinator relationships in future studies applying pollination syndromes.

Studies based on the classical, categorical definitions of pollination syndromes, such as the presence or absence of nectar or long versus short corolla tubes [16,25,29] found only weak support for pollination syndromes. Conversely, studies developing the classical concept further, using quantitative trait data [18,55] or data on pollination networks [21,33] strongly supported the concept. Our direct comparison of quantitative and categorical data of the same set of species and traits (Fig 3) thus demonstrates that the low support of the pollination syndrome concept found when categorical data are used is likely due to a reduced data resolution. Consequently, this weakens the conclusions obtained from studies using only categorical data [28]. However, Ollerton´s et al. [28,29] hypothesis that the accuracy of the syndrome predictions differs across plant families might still hold: All studies, using quantitative trait data [18,22,34,55] investigated plant groups with complex, zygomorphic flowers (e.g., South African species with mostly complex flower, Balsaminaceae, Calceolariaceae, Gesneriaceae, Lamiaceae), likely more highly adapted to specific guilds of pollinators.

## Conclusion

The present study is able to demonstrate the presence of classically defined pollination syndromes in Balsaminaceae based on flower morphometry, and quantified signal and reward data. Similar results have been obtained for several other plant groups with complex flower architectures. It remains to be seen whether less complex, e.g., radial, symmetrical flowers, will be amenable to the type of analysis here performed for florally complex Balsaminaceae. The results of such studies will strongly depend on data quality (categorical vs. quantitative data) as well as on the specific traits used. Data are only informative if they capture the functionally relevant dimensions of plant-pollinator relationships, i.e., the ones that actually determine effective flower visitation in the natural habitat. If the chosen characters correctly characterize the flower, but fail to reflect its functional aspects, then pollination syndromes can not be teased out by means of statistical analysis, no matter how sophisticated.

## Supporting information

S1 Fig Clustering received from multiscale bootstrap resampling. Numbers represent bootstrap support of the backbone only. Colors represent pollination syndromes.
(DOC)

S2 Fig Kruskal-Wallis tests for multiple comparisons of several flower morphometry, signal and rewards traits between different pollination syndromes defined by the cluster analysis. Note that Large bee 1 (= *Impatiens glandulifera*) and Moth (= *I*. *sodenii*) was not included into the analyses due to too small sampling sizes.
(DOC)

S1 Table Analysed species with accession and herbarium numbers–these numbers are identical since we took herbarium specimen only from accessions of the Botanical Gardens Bonn—as well as morphometry and reward traits. Note that the large number of unidentified *Impatiens* species is due to the large number of undescribed species in this genus (pers. obs. E. Fischer).
(DOC)

S2 Table Pollinator observations in the natural habitat for the studied Balsaminaceae species. (DOC)

S3 Table Definitions for the categorisation of the flower morphometry, signal and reward traits. (DOC)

S4 Table Results of the Spearman correlation analyses between flower morphometry, signal and reward traits. (DOC)
