# Supplementary material for: A question of data quality—Testing pollination syndromes in Balsaminaceae
Source: PLoS One. 2017 Oct 16;12(10):e0186125. doi: 10.1371/journal.pone.0186125 (PMC5642891; doi:10.1371/journal.pone.0186125)
Supplement: S1 Fig — Numbers represent bootstrap support of the backbone only. Colors represent pollination syndromes. (DOC) [file pone.0186125.s001.doc]

**S1 Figure:** Clustering received from multiscale bootstrap resampling. Numbers represent bootstrap support of the backbone only. Colors represent pollination syndromes.
