# Supplementary material for: A question of data quality—Testing pollination syndromes in Balsaminaceae
Source: PLoS One. 2017 Oct 16;12(10):e0186125. doi: 10.1371/journal.pone.0186125 (PMC5642891; doi:10.1371/journal.pone.0186125)
Supplement: S2 Fig — Note that Large bee 1 (= Impatiens glandulifera) and Moth (= I. sodenii) was not included into the analyses due to too small sampling sizes. (DOC) [file pone.0186125.s002.doc]

**S2 Figure:** Kruskal-Wallis tests for multiple comparisons of several flower morphometry, signal and rewards traits between different pollination syndromes defined by the cluster analysis. Note that Large bee 1 (= *Impatiens glandulifera*) and Moth (= *I. sodenii*) was not included into the analyses due to too small sampling sizes.
