# Supplementary material for: A question of data quality—Testing pollination syndromes in Balsaminaceae
Source: PLoS One. 2017 Oct 16;12(10):e0186125. doi: 10.1371/journal.pone.0186125 (PMC5642891; doi:10.1371/journal.pone.0186125)
Supplement: S1 Table — Note that the large number of unidentified Impatiens species is due to the large number of undescribed species in this genus (pers. obs. E. Fischer). (DOC) [file pone.0186125.s003.doc]

**S1 Table:** Analysed species with accession and herbarium numbers – these numbers are identical since we took herbarium specimen only from accessions of the Botanical Gardens Bonn - as well as morphometry and reward traits. Note that the large number of unidentified *Impatiens* species is due to the large number of undescribed species in this genus (pers. obs. E. Fischer).

|  | **Accession &**  **herbarium no.** | **Spur**  **length (mm)** | **Spur-carrying sepal length (mm)** | **Total flower**  **length (mm)** | **Flower opening width (mm)** | **Total flower**  **width (mm)** | **Dorsal petal**  **length (mm)** | **Nectar**  **volume (μL)** | **Sugar**  **concentration (%)** | **Sugar**  **amount (mg)** | **Display**  **size total (cm²)** | **Display**  **size frontal (cm²)** | **Display size lateral (cm²)** | **Display size frontal/lateral** |
| --- | --- | --- | --- | --- | --- | --- | --- | --- | --- | --- | --- | --- | --- | --- |
| *Hydrocera triflora* | 35302 | 7.0 | 6.92 | 26.28 | 7.96 | 11.64 | 16.44 | 1.18 | 34.32 | 0.46 | 4.05 | 2.09 | 1.96 | 1.06 |
| *I*. aff. *catatii* | 28424 | 10.1 | 20.5 | 32.2 | 4.25 | 13.3 | 20.5 | 10.09 | 28.58 | 3.26 | 4.47 | 1.95 | 2.53 | 0.77 |
| *I.* aff. *congolensis* | 30441 | 8.6 | 24.6 | 48.4 | 3.8 | 12.8 | 17.2 | 31.95 | 22.88 | 7.95 | 5.54 | 2.12 | 3.42 | 0.62 |
| *I.* aff. *inaperta* | 37668 | 0.0 | 0.0 | 2.25 | 1.4 | 2.6 | 2.6 | 0.0 | 0.0 | 0 | 0.02 | 0.01 | 0.01 | 0.87 |
| *I.* aff. *sambiranensis* | 36428 | 29.07 | 4.79 | 47.43 | 1.62 | 26.14 | 35.43 | 1.09 | 25.33 | 0.3 | 6.19 | 4.83 | 1.36 | 3.55 |
| *I. arguta* | 36166 | 10.55 | 16.91 | 44.0 | 7.91 | 18.73 | 7.91 | 6.56 | 41.32 | 3.18 | 8.56 | 3.63 | 4.93 | 0.74 |
| *I. auricoma* | 34154 | 4.33 | 7.8 | 19.2 | 1.8 | 9.0 | 10.6 | 5.5 | 24.16 | 1.45 | 5.68 | 2.39 | 3.29 | 0.73 |
| *I. balfourii* | 36126 | 18.92 | 8.76 | 36.68 | 11.48 | 30.3 | 35.9 | 4.06 | 45.44 | 2.2 | 5.85 | 3.6 | 2.25 | 1.6 |
| *I. balsamina* | 36195 | 17.3 | 6.1 | 39.8 | 5.2 | 34.7 | 30.8 | 3.38 | 34.8 | 1.36 | 6.96 | 4.75 | 2.21 | 2.15 |
| *I. bequaertii* | 36126 | 6.0 | 1.1 | 10.7 | 0.23 | 11.0 | 12.7 | 0.34 | 24.24 | 0.09 | 0.86 | 0.57 | 0.29 | 1.97 |
| *I. bicolor* | 33521 | 11.8 | 19.7 | 30.7 | 3.1 | 12.45 | 22.0 | 14.39 | 22.94 | 3.58 | 3.74 | 1.57 | 2.17 | 0.72 |
| *I. bisaccata* | 36496 | 3.4 | 5.6 | 18.8 | 1.9 | 19.0 | 35.8 | 1.5 | 32.32 | 0.54 | 6.9 | 4.35 | 2.56 | 1.7 |
| *I. bombycina* | 36143 | 13.6 | 27.5 | 54.1 | 3.1 | 10.8 | 21.6 | 36.03 | 21.56 | 8.64 | 4.23 | 1.99 | 2.24 | 0.88 |
| *I. briartii* | 36135 | 25.9 | 5.5 | 40.1 | 0.95 | 15.0 | 22.5 | 0.45 | 16.96 | 0.08 | 2.16 | 1.32 | 0.84 | 1.57 |
| *I. burtonii* | 11528 | 10.1 | 5.5 | 17.2 | 3.3 | 20.2 | 25.0 | 0.39 | 39.44 | 0.18 | 5.81 | 3.52 | 2.29 | 1.54 |
| *I. bururiensis* | 36564 | 13.4 | 23.8 | 47.6 | 4.2 | 10.1 | 19.7 | 51.08 | 24.36 | 13.5 | 4.77 | 2.11 | 2.66 | 0.79 |
| *I. campanulata* | 36242 | 5.5 | 4.8 | 20.6 | 5.2 | 15.6 | 24.1 | 2.83 | 44.91 | 1.53 | 3.18 | 1.49 | 1.69 | 0.88 |
| *I. cinnabarina* | 34145 | 35.57 | 5.0 | 55.4 | 1.0 | 30.52 | 33.0 | 6.38 | 22.92 | 1.59 | 5.92 | 4.58 | 1.34 | 3.42 |
| *I. clavicalcar* | 27465 | 7.8 | 17.6 | 27.7 | 3.4 | 10.6 | 21.8 | 11.0 | 24.62 | 3 | 4.8 | 1.98 | 2.82 | 0.7 |
| *I. columbaria* | 13781 | 5.55 | 5.18 | 18.0 | 1.0 | 19.82 | 24.91 | 0.81 | 42.78 | 0.42 | 2.79 | 1.51 | 1.28 | 1.18 |
| *I. confusa* | 27471 | 33.3 | 3.3 | 47.7 | 1.2 | 27.4 | 23.7 | 4.58 | 19.14 | 0.94 | 4.35 | 3.18 | 1.17 | 2.7 |
| *I. congolensis* | 31069 | 8.4 | 25.7 | 38.8 | 3.8 | 11.9 | 24.6 | 32.37 | 23.44 | 8.05 | 5.78 | 1.95 | 3.83 | 0.51 |
| *I. cordata* | 36127 | 17.8 | 3.1 | 30.1 | 0.95 | 5.05 | 24.3 | 1.85 | 27.4 | 0.55 | 7.32 | 6.21 | 1.11 | 5.6 |
| *I. elatostemmoides* | 26821 | 0.0 | 0.0 | 2.8 | 0.2 | 4.95 | 4.5 | 0.03 | 0.0 | 0 | 0.12 | 0.09 | 0.03 | 3.08 |
| *I. elianae* | 36144 | 43.9 | 6.0 | 60.5 | 1.0 | 24.8 | 25.8 | 4.1 | 31.96 | 1.48 | 4.16 | 3.07 | 1.09 | 2.81 |
| *I. erecticornis* | 32579 | 22.6 | 2.5 | 29.6 | 1.5 | 29.6 | 30.5 | 4.8 | 20.46 | 1.04 | 8.72 | 5.32 | 3.4 | 1.57 |
| *I. eriosperma* | 35921 | 10.3 | 18.5 | 42.1 | 2.9 | 14.0 | 21.0 | 24.32 | 32.92 | 9.12 | 4.26 | 2.69 | 1.57 | 1.72 |
| *I. ethiopica* | 36142 | 17.3 | 4.3 | 29.9 | 1.9 | 21.4 | 21.0 | 1.65 | 29.6 | 0.56 | 3.59 | 1.43 | 2.16 | 0.66 |
| *I. flaccida alba* | 36244 | 35.4 | 6.5 | 55.3 | 1.0 | 37.7 | 38.7 | 4.38 | 31.48 | 1.53 | 11.89 | 8.81 | 3.09 | 2.85 |
| *I. gesneroidea* | 32578 | 16.5 | 15.2 | 39.7 | 3.9 | 10.8 | 12.7 | 26.02 | 28.24 | 8.14 | 2.83 | 1.31 | 1.52 | 0.87 |
| *I. glandulifera* | 28969 | 8.3 | 21.3 | 29.6 | 8.5 | 31.3 | 48.7 | 5.1 | 63.56 | 4.28 | 10.34 | 4.76 | 5.58 | 0.85 |
| *I. hammarbyoides* | 36433 | 0.0 | 0.0 | 2.5 | 1.5 | 1.0 | 2.5 | 0.0 | 0.0 | 0 | 0.02 | 0.01 | 0.01 | 1.12 |
| *I. hians* | 5606 | 7.0 | 35.6 | 57.3 | 8.8 | 12.8 | 33.2 | 25.11 | 22.32 | 6.02 | 5.38 | 2.98 | 2.4 | 1.24 |
| *I. hochstetteri* | 17872 | 14.8 | 2.7 | 18.8 | 0.72 | 14.3 | 16.4 | 0.08 | 27.79 | 0.03 | 0.83 | 0.6 | 0.24 | 2.54 |
| *I. humillima* | 36426 | 0.0 | 0.0 | 3.6 | 0.73 | 3.2 | 6.1 | 0.0 | 0.0 | 0 | 0.07 | 0.04 | 0.02 | 1.71 |
| *I. inaperta* | 27467 | 0.0 | 0.0 | 1.5 | 1.67 | 2.33 | 3.0 | 0.0 | 0.0 | 0 | 0.04 | 0.02 | 0.02 | 0.94 |
| *I. keilii* | 17556 | 7.6 | 22.1 | 29.5 | 3.6 | 6.3 | 12.5 | 5.85 | 22.2 | 1.4 | 3.13 | 1.29 | 1.85 | 0.7 |
| *I. kilimanjari* | 17989 | 5.5 | 19 | 33.5 | 3.0 | 14.8 | 16.1 | 8.13 | 20.28 | 1.76 | 2.45 | 1.21 | 1.23 | 0.98 |
| *I. kinabaulensis* | 34121 | 27.4 | 3.4 | 42.9 | 1.0 | 32.8 | 25.7 | 1.15 | 22.04 | 0.28 | 4.5 | 3.69 | 0.81 | 4.56 |
| *I. lateriflora* | 36131 | 31.0 | 4.8 | 53.8 | 7.2 | 43.1 | 56.7 | 3.85 | 26.96 | 1.15 | 17.48 | 12.58 | 4.9 | 2.57 |
| *I. laurentii* | 36132 | 45.3 | 6.4 | 67.0 | 4.5 | 33.2 | 36.7 | 2.37 | 23.32 | 0.59 | 8.44 | 6.29 | 2.14 | 2.94 |
| 1. *mackeyana*   ssp. *zenkeri* | 6550 | 11.4 | 12.5 | 39.2 | 9.5 | 22.1 | 29.9 | 7.54 | 35.5 | 3.14 | 8.77 | 4.23 | 4.54 | 0.93 |
| *I. mandrakae* | 26822 | 0.0 | 0.0 | 4.15 | 0.32 | 4.8 | 9.9 | 0.02 | 0.0 | 0 | 0.29 | 0.21 | 0.08 | 2.58 |
| *I. masoalensis* | 36386 | 5.2 | 2.9 | 15.1 | 2.0 | 9.3 | 19.8 | 0.59 | 36.71 | 0.25 | 2.64 | 1.92 | 0.72 | 2.69 |
| *I. mishmiensis* | 36651 | 16.1 | 24.3 | 53.3 | 8.4 | 16.0 | 29.4 | 9.8 | 40.48 | 4.61 | 8.39 | 4.38 | 4.01 | 1.09 |
| *I. morsei* | 28039 | 7.0 | 16.0 | 41.25 | 14.0 | 29.42 | 36.0 | 6.11 | 31.24 | 2.13 | 9.76 | 3.89 | 5.87 | 0.66 |
| *I. namchabarwensis* | 17994 | 13.18 | 10.35 | 35.06 | 6.12 | 26.41 | 33.88 | 1.3 | 41.3 | 0.61 | 7.15 | 4.13 | 3.02 | 1.37 |
| *I. niamniamensis 10* | 10305 | 13.0 | 23.3 | 34.8 | 3.0 | 12.2 | 28.0 | 50.15 | 19.32 | 10.3 | 4.69 | 1.74 | 2.95 | 0.59 |
| *I. niamniamensis 11* | 11521 | 15.7 | 26.6 | 36.1 | 3.0 | 10.7 | 24.0 | 45.3 | 20.9 | 10.2 | 7.82 | 3.0 | 4.82 | 0.62 |
| *I. niamniamensis 31* | 31233 | 10.29 | 32.29 | 54.14 | 3.36 | 8.5 | 18.71 | 32.09 | 25.5 | 9.25 | 5.69 | 1.99 | 3.7 | 0.54 |
| *I. noli-tangere* | 33527 | 7.4 | 17.2 | 30.6 | 5.7 | 10.3 | 24.3 | 2.98 | 46.76 | 1.7 | 3.24 | 1.59 | 1.65 | 0.97 |
| *I. nyungwensis* | 18406 | 16.3 | 7.65 | 32.5 | 3.62 | 17.6 | 19.1 | 9.63 | 24.52 | 2.63 | 2.96 | 1.27 | 1.69 | 0.75 |
| *I. parasitica* | 28428 | 5.3 | 17.2 | 24.5 | 2.95 | 9.1 | 11.0 | 12.64 | 24.12 | 3.33 | 2.73 | 1.2 | 1.53 | 0.78 |
| *I. parviflora* | 36893 | 4.0 | 4.3 | 14.1 | 2.15 | 6.6 | 13.2 | 0.06 | 44.28 | 0.03 | 1.27 | 0.74 | 0.54 | 1.37 |
| *I. paucidentata* | 4819 | 10.6 | 31.3 | 41.5 | 4.7 | 11.4 | 17.3 | 16.07 | 15.0 | 2.55 | 6.96 | 3.79 | 3.18 | 1.19 |
| *I. pinganoensis* | 36626 | 11.7 | 3.0 | 21.9 | 0.9 | 12.4 | 13.8 | 0.56 | 24.24 | 0.15 | 1.73 | 1.18 | 0.55 | 2.14 |
| *I. platypetala* | 36569 | 38.6 | 6.0 | 39.15 | 1.6 | 38.55 | 43.75 | 5.38 | 30.0 | 1.82 | 7.28 | 5.88 | 1.4 | 4.2 |
| *I. poilanei* | 34147 | 34.52 | 7.9 | 60.0 | 1.0 | 21.6 | 27.6 | 2.9 | 26.44 | 0.84 | 2.95 | 1.84 | 1.11 | 1.66 |
| *I. pseudoviola* | 36595 | 19.4 | 2.9 | 30.7 | 0.9 | 20.9 | 22.3 | 1.26 | 29.0 | 0.41 | 3.89 | 3.05 | 0.84 | 3.63 |
| *I. puberbula* | 36758 | 11.0 | 7.4 | 27.2 | 2.8 | 11.6 | 29.6 | 12.51 | 20.8 | 2.14 | 6.16 | 4.05 | 2.11 | 1.92 |
| *I. purpureoviolacea* | 12079 | 22.3 | 4.2 | 27.3 | 1.94 | 24.3 | 35.0 | 1.99 | 24.16 | 2.81 | 6.09 | 3.85 | 2.24 | 1.72 |
| *I. pyschadelphoides* | 28425 | 35.0 | 5.0 | 51.3 | 4.9 | 15.7 | 40.3 | 5.5 | 33.68 | 0.52 | 9.3 | 7.83 | 1.47 | 5.32 |
| *I. rutenbergii* | 27463 | 37.16 | 12.0 | 67.3 | 3.64 | 41.09 | 35.7 | 9.0 | 28.72 | 2.91 | 5.73 | 3.37 | 2.36 | 1.43 |
| *I. scabrida* | 11186 | 10.7 | 12.9 | 37.5 | 5.2 | 22.1 | 32.6 | 4.06 | 49.16 | 2.44 | 10.0 | 4.66 | 5.34 | 0.87 |
| *I. sodenii* | 28429 | 128.42 | 11.0 | 146.89 | 0.96 | 48.89 | 55.3 | 8.81 | 10.94 | 1.01 | 26.19 | 19.54 | 6.65 | 2.94 |
| *I.* spec. 06 | 17206 | 15.6 | 1.7 | 24.6 | 1.5 | 17.8 | 26.6 | 2.05 | 23.42 | 0.51 | 3.83 | 2.52 | 1.31 | 1.92 |
| *I.* spec. 20 | 34120 | 32.8 | 3.3 | 51.2 | 1.0 | 33.0 | 32.7 | 32.37 | 26.2 | 9.33 | 5.79 | 4.53 | 1.26 | 3.6 |
| *I.* spec. 40 | 36240 | 18.24 | 3.4 | 29.3 | 1.0 | 22.3 | 25.6 | 4.57 | 27.72 | 1.43 | 17.93 | 17.22 | 0.71 | 24.26 |
| *I.* spec. 48 | 36248 | 32.5 | 6.6 | 52.3 | 2.0 | 13.0 | 47.2 | 4.67 | 22.72 | 1.16 | 14.7 | 10.48 | 4.21 | 2.49 |
| *I.* spec. 55 | 34555 | 36.6 | 7.6 | 55.2 | 1.0 | 55.3 | 52.1 | 19.08 | 27.52 | 5.97 | 13.29 | 10.26 | 3.03 | 3.39 |
| *I.* spec. 56 | 34556 | 35.0 | 6.6 | 52.5 | 1.0 | 48.3 | 46.4 | 20.27 | 27.64 | 6.34 | 12.53 | 9.62 | 2.91 | 3.31 |
| *I.* spec. 57 | 34557 | 7.9 | 2.3 | 18.3 | 1.2 | 14.4 | 26.3 | 0.94 | 21.8 | 0.23 | 2.07 | 1.48 | 0.59 | 2.52 |
| *I.* spec. 58 | 34558 | 14.85 | 3.69 | 25.0 | 1.0 | 17.31 | 33.8 | 1.04 | 29.0 | 0.34 | 3.17 | 1.95 | 1.22 | 1.6 |
| *I.* spec. 59 | 36659 | 20.9 | 13.8 | 43.0 | 4.7 | 16.6 | 23.8 | 3.28 | 41.28 | 1.59 | 3.51 | 1.86 | 1.65 | 1.13 |
| *I.* spec. 70 | 35170 | 30.2 | 8.1 | 51.8 | 1.16 | 32.1 | 44.5 | 2.66 | 25.56 | 0.77 | 10.83 | 8.35 | 2.49 | 3.36 |
| *I.* spec. 72 | 27472 | 68.3 | 9.9 | 88.2 | 1.8 | 24.6 | 30.1 | 15.91 | 25.72 | 4.58 | 10.4 | 6.38 | 4.03 | 1.58 |
| *I.* spec. 86 | 33486 | 9.0 | 7.32 | 25.1 | 1.2 | 18.28 | 26.75 | 1.53 | 26.76 | 0.46 | 5.38 | 3.62 | 1.76 | 2.06 |
| *I. stenantha* | 36164 | 13.1 | 10.0 | 31.0 | 3.9 | 13.1 | 15.8 | 2.42 | 44.32 | 1.27 | 1.47 | 0.65 | 0.82 | 0.79 |
| *I. stuhlmannii* | 16583 | 34.8 | 4.9 | 52.2 | 1.0 | 41.9 | 44.9 | 8.33 | 24.16 | 2.2 | 14.53 | 8.19 | 6.34 | 1.29 |
| *I. teitensis* | 27473 | 46.6 | 5.2 | 53.6 | 1.9 | 48.1 | 39.5 | 10.77 | 22.88 | 2.68 | 18.24 | 13.76 | 4.48 | 3.07 |
| 1. *teitensis*   ssp. *teitensis* | 36189 | 61.04 | 5.2 | 75.4 | 1.2 | 52.56 | 52.78 | 10.07 | 29.04 | 3.25 | 12.1 | 10.62 | 1.48 | 7.17 |
| *I. uniflora* | 36165 | 12.1 | 12.6 | 34.6 | 6.0 | 10.3 | 18.89 | 7.22 | 38.12 | 3.2 | 3.77 | 1.38 | 2.39 | 0.58 |
| *I. trichoceras* | 152 | 65.9 | 8.9 | 67.7 | 1.03 | 29.0 | 31.6 | 12.27 | 29.48 | 3.96 | 9.49 | 6.18 | 3.31 | 1.87 |
| *I. usambarensis* | 13386 | 24.8 | 4.9 | 46.9 | 1.0 | 45.8 | 45.5 | 3.35 | 36.6 | 1.44 | 12.33 | 12.07 | 0.26 | 46.42 |
| *I. walleriana* | 36585 | 24.8 | 3.6 | 42.0 | 0.74 | 39.6 | 37.3 | 2.11 | 37.68 | 0.94 | 5.06 | 4.29 | 0.77 | 5.6 |
| *I. warburgiana* | 32580 | 19.5 | 2.24 | 26.34 | 1.3 | 16.4 | 12.4 | 1.51 | 30.6 | 0.53 | 2.62 | 1.29 | 1.33 | 0.97 |
