# Supplementary material for: A question of data quality—Testing pollination syndromes in Balsaminaceae
Source: PLoS One. 2017 Oct 16;12(10):e0186125. doi: 10.1371/journal.pone.0186125 (PMC5642891; doi:10.1371/journal.pone.0186125)
Supplement: S2 Table — (DOC) [file pone.0186125.s004.doc]

**S2 Table:** Pollinator observations in the natural habitat for the studied Balsaminaceae species.

| ***Impatiens* species** | **Pollinator records** | **Reference** |
| --- | --- | --- |
| *Impatiens balfourii* | Bees (*Apis mellifera, Bombus hortorum, B. pascourum*) | Ugoletti *et al.* 2013 |
| *Impatiens balsamina* | Butterflies (*Papilio protenor*) | Suzuki *et al.* 1987 |
| *Impatiens bisaccata* | Bees (*Pachymelus unicolor, P. bicolor, P. cambouii*) | Erpenbach 2006 |
| *Impatiens burtonii* | Bees, syrphid flies | Vlašánková *et al*. 2017 |
| *Impatiens campanulata* | Bees (*Apis cerana, Trigona* spec.*);* butterflies *(Macroglossum variegatum, M. corythus*) | Kulloli *et al.* 2009 |
| *Impatiens* aff. *catatii* | Sunbirds (*Nectarinia sovimanga*) | Erpenbach 2006 |
| *Impatiens elatostemmoides* | Flies (Brachycera), microhymenoptera | Erpenbach 2006 |
| *Impatiens glandulifera* | Bees (*Bombus agrorum, B. terrestris, B. lapidarius, B. keriensis, B. asiaticus, B. rufofasiatus, B. tunicatus, Apis mellifera*) | Knuth 1906 (in Europe); Saini & Ghattor 2007 (in the Himalaya) |
| *Impatiens hians* | Sunbirds (*Cyanomitra obscura, C. oritis*) | Janeček *et al.* 2015 |
| *Impatiens hochstetteri* | Flies (*Stenobasipteron wiedemanni*), papilioid butterfly; butterflies (*Papilio echerioides, P. nireus, P. demodocus*) | Vogel 1954; [Potgieter & Edwards 2005](http://www.ispot.org.za/node/183157?nav=related; 02.12.2014) |
| *Impatiens kilimanjari* | Sunbirds (*Cyanomitra olivacea, Nectarinia mediocris*) | Maximilian Vollstaedt (pers comm.) |
| *Impatiens mandrakae* | Mosquitos (Nematocera), microhymenoptera | Erpenbach 2006 |
| *Impatiens niamniamensis* (red-white flowers)  *Impatiens niamniamensis* (red-yellow flowers) | Sunbirds (*Cyanomitra oritis; C. alinae*)  Sunbirds | Janeček *et al.* 2015  Grey-Wilson 1980; Cheke & Mann 2008 |
| *Impatiens noli-tangere* | Bees (*Bombus diversus, B. lapidarius, hortorum, B. terrestris*) | Kato 1988 |
| *Impatiens parviflora* | Flies (*Episyrphus balteatus, Melanostoma mellinum, Platycheirus albimanus, Rhingia campestris, Syrphus balteatus, S. corollae, S. ribesii*) | Vervoort *et al.* 2011; Knuth 1906 |
| *Impatiens pinganoensis* | Flies (*Empis* spec.) | Abrahamczyk *et al.* 2016 |
| *Impatiens platypetala* | Butterflies (*Macroglossum corythus*) | Kato *et al.* 1991 |
| *Impatiens scabrida* | Bees (*Bombus* spp*.*) | Kumar & Lall 1998 |
| *Impatiens usambarensis* | Butterflies | Grey-Wilson 1980 |
| *Impatiens walleriana* | Butterflies | Vogel 1954; Grey-Wilson 1980 |
|  |  |  |
| **Pollinator observations**  **Bonn, Aug./Sept. 2017**  *Impatiens arguta* | **in the Botanical Gardens**  Bees (*Bombus pascuorum*) | pers. obs. S. Abrahamczyk |

Abrahamczyk, S., Janssens, S., Xixima, L., Ditsch, B. & Fischer, E. (2016) *Impatiens*

*pinganoensis* (Balsaminaceae), a new species from Angola. Phytotaxa 261: 240–250.

Erpenbach, A. (2006). Blütenökologie madagassischer Springkräuter (*Impatiens*,

Balsaminaceae). Diploma thesis, University of Bonn.

Janeček, Š., Bartoš, M. & Njabo, K.Y. (2015). Convergent evolution of sunbird pollination

systems of *Impatiens* species in tropical Africa and hummingbird systems of the New

World. Biological Journal of the Linnean Society 115: 127-133.

Cheke, R. & Mann, C. (2008). Family Nectariniidae (Sunbirds) In: Handbook of the bird

families of the world. Vol. 8. Lynx Editions, Barcelona.

Grey-Wilson, C. (1980). *Impatiens* of Africa. Balkema, P. O. Bos 1675, Rotterdam,

Netherlands.

Kato, M., Ichino, T., Hotta, M. & Inoue, T. (1991). Pollination of four Sumatran *Impatiens*

species by hawkmoths and bees. Tropics 1: 59-73.

Knuth, P. (1906). 1898–1905. Handbuch der Blütenbiologie. Vol. I-III Wilhelm Engelmann

Verlag, Leipzig.

Kulloli, S.K., Ramasubbu, R., Sreekala, A.K. & Pandurangan, A.G. (2009). Reproductive

ecology of *Impatiens campanulata* Wight – a rare and endemic balsam of southern

Western Ghats. Ecology, Environment and Conservation 15: 235-239.

Kumar, A. & Lall, A. (1998). Bumble bee species and flowering plant relationships at high

altitude in north west India. Indian Journal of Ecology 25: 1-7.

Potgieter, C. J., & Edwards, T. J. (2005). The *Stenobasipteron wiedemanni* (Diptera,

Nemestrinidae) pollination guild in eastern southern Africa. Annals of the Missouri

Botanical Garden 92: 254-267.

Saini, M.S., & Ghattor, H.S. (2007). Taxonomy and food plants of some bumble bee species

of Lahaul and Spiti valley of Himachal Pradesh. Zoos' Print Journal *22*: 2648-2657.

Suzuki, N., Yamashita, K., Niizuma, A. & Kiritani, K. (1987). Studies on ecology and

behavior of Japanese black swallowtail butterflies. 6. Nectar feeding of *Papilio*

*helenus nicconicolens* Butler and *P. protenor demetrius* Cramer as main pollinators of

glory bower, *Clerodendron trichotomum*, Thunb. *Ecological Research*, 2, 41-52.

Ugoletti, P., Reidy, D., Jones, M. B. & Stout, J.C. (2013). Do native bees have the potential

to promote interspecific pollination in introduced *Impatiens* species? *Journal of*

*Pollination Ecology*, 11, 1-8.

Vlašánková, A., Padyšáková, E., Bartoš, M., Mengual, X., Janečková, P., & Janeček, Š.

(2017). The nectar spur is not only a simple specialization for long-proboscid

pollinators. *New Phytologist*, doi: 10.1111/nph.14677.

Vogel, S. (1954). *Blütenbiologische Typen als Elemente der Sippengliederung:*

*dargestellt anhand der Flora Südafrikas*. G. Fischer Jena.
