# Supplementary material for: A question of data quality—Testing pollination syndromes in Balsaminaceae
Source: PLoS One. 2017 Oct 16;12(10):e0186125. doi: 10.1371/journal.pone.0186125 (PMC5642891; doi:10.1371/journal.pone.0186125)
Supplement: S3 Table — (DOC) [file pone.0186125.s005.doc]

**S3 Table:** Definitions for the categorisation of the flower morphometry, signal and reward traits.

|  | **A** | **B** | **C** |
| --- | --- | --- | --- |
| **Spur length (mm)** | 0.00 | 1.00-19.99 | 20.00-128.42 |
| **Spur-carrying sepalum (mm)** | 0.00 | 0.01-6.99 | 7.00-35.60 |
| **Total flower length (mm)** | 0.01-29.90 | 30.00-146.89 | - |
| **Flower opening width (mm)** | 0.01-2.49 | 2.50-14.00 | - |
| **Total width (mm)** | 0.01-19.99 | 20.00-55.30 | - |
| **Dorsal petal length (mm)** | 0.01-24.99 | 25.00-52.10 | - |
| **Nectar volume (μL)** | 0.00 | 0.01-4.99 | 5.00-51.08 |
| **Sugar concentration (%)** | 0.00 | 0.01-29.99 | 30.00-63.56 |
| **Sugar amount (mg)** | 0.00 | 0.01-1.99 | 2.00-12.79 |
| **Display size total (cm²)** | 0.01-4.99 | 5.00-26.19 | - |
| **Display size frontal (cm²)** | 0.01-2.99 | 3.00-19.54 | - |
| **Display size lateral (cm²)** | 0.01-1.99 | 2.00-6.65 | - |
| **Display size frontal/lateral** | 0.01-1.99 | 2.00-46.42 | - |
