# Supplementary material for: A question of data quality—Testing pollination syndromes in Balsaminaceae
Source: PLoS One. 2017 Oct 16;12(10):e0186125. doi: 10.1371/journal.pone.0186125 (PMC5642891; doi:10.1371/journal.pone.0186125)
Supplement: S4 Table — (DOC) [file pone.0186125.s006.doc]

**S4 Table:** Results of the Spearman correlation analyses between flower morphometry, signal and reward traits.

|  | Spur  length | Sepal  length | Total  flower length | Opening | Total  flower width | Dorsal petal  length | Nectar  volume | Sugar  concentration | Sugar  amount | Display size  total | Display size  frontal | Display size  lateral |
| --- | --- | --- | --- | --- | --- | --- | --- | --- | --- | --- | --- | --- |
| Sepal length | R = -0.02  p = 0.863 |  |  |  |  |  |  |  |  |  |  |  |
| Total length | R = 0.77  **p < 0.001** | R = 0.45  **p < 0.001** |  |  |  |  |  |  |  |  |  |  |
| Opening | R = -0.21  p = 0.054 | R = 0.65  **p < 0.001** | R = 0.134  p = 0.220 | |  |  |  |  |  |  |  |  |
| Total width | R = 0.75  **p < 0.001** | R = -0.06  p = 0.604 | R = 0.59  **p < 0.001** | R = -0.10  p = 0.383 | |  |  |  |  |  |  |  |
| Front length | R = 0.66  **p < 0.001** | R = 0.08  p = 0.471 | R = 0.59  **p < 0.001** | R = 0.03  p = 0.817 | R = 0.82  **p < 0.001** |  |  |  |  |  |  |  |
| Nectar amount | R = 0.29  **p = 0.007** | R = 0.75  **p < 0.001** | R = 0.61  **p < 0.001** | R = 0.38  **p < 0.001** | R = 0.14  p = 0.202 | R = 0.23  **p = 0.033** |  |  |  |  |  |  |
| Sugar  concentration | R = 0.08  p = 0.475 | R = 0.15  p = 0.171 | R = 0.06  p = 0.562 | R = 0.38  **p < 0.001** | R = 0.30  **p = 0.005** | R = 0.24  **p = 0.024** | R = -0.07  p = 0.54 |  |  |  |  |  |
| Sugar amount | R = 0.25  **p = 0.021** | R = 0.79  **p < 0.001** | R = 0.59  **p < 0.001** | R = 0.47  **p < 0.001** | R = 0.14  p = 0.174 | R = 0.24  **p = 0.025** | R = 0.97  **p < 0.001** | R = 0.11  p = 0.317 |  |  |  |  |
| Display size total | R = 0.59  **p < 0.001** | R = 0.29  **p = 0.008** | R = 0.64  **p < 0.001** | R = 0.18  p = 0.100 | R = 0.67  **p < 0.001** | R = 0.79  **p < 0.001** | R = 0.47  **p < 0.001** | R = 0.19  p = 0.078 | R = 0.46  **p < 0.001** |  |  |  |
| Display size  frontal | R = 0.68  **p < 0.001** | R = 0.11  p = 0.292 | R = 0.65  **p < 0.001** | R = 0.03  p = 0.769 | R = 0.75  **p < 0.001** | R = 0.85  **p < 0.001** | R = 0.34  **p = 0.001** | R = 0.20  p = 0.065 | R = 0.32  **p = 0.002** | R = 0.96  **p < 0.001** | |  |
| Display size  lateral | R = 0.23  **p = 0.035** | R = 0.66  **p < 0.001** | R = 0.49  **p < 0.001** | R = 0.54  **p < 0.001** | R = 0.32  **p = 0.003** | R = 0.44  **p < 0.001** | R = 0.64  **p < 0.001** | R = 0.14  p = 0.189 | R = 0.64  **p < 0.001** | R = 0.71  **p < 0.001** | R = 0.53  **p < 0.001** |  |
| Display size  frontal/lateral | R = 0.52  **p < 0.001** | R = -0.58  **p < 0.001** | R = 0.22  **p = 0.046** | R = -0.59  **p < 0.001** | R = 0.50  **p < 0.001** | R = 0.48  **p < 0.001** | R = -0.30  **p = 0.005** | R = -0.02  p = 0.879 | R = -0.34  **p = 0.001** | R = 0.29  **p = 0.007** | R = 0.51  **p < 0.001** | R = -0.38  **p < 0.001** |
